# A Label-Based Polymer Nanoparticles Biosensor Combined with Loop-Mediated Isothermal Amplification for Rapid, Sensitive, and Highly Specific Identification of *Brucella abortus*

**DOI:** 10.3389/fbioe.2021.758564

**Published:** 2021-11-18

**Authors:** Xinggui Yang, Yue Wang, Ying Liu, Junfei Huang, Qinqin Tan, Xia Ying, Yong Hu, Shijun Li

**Affiliations:** ^1^ Guizhou Provincial Center for Disease Control and Prevention, Guiyang, China; ^2^ School of Public Health, the Key Laboratory of Environmental Pollution Monitoring and Disease Control, Ministry of Education, Guizhou Medical University, Guiyang, China

**Keywords:** *Brucella abortus*, nanoparticles biosensor, loop-mediated isothermal amplification, BruAb2_0168 gene, blood

## Abstract

*Brucella abortus* (*B. abortus*), an important zoonotic pathogen in *Brucella* spp., is the major causative agent of abortion in cattle (namely, bovine brucellosis). Currently, although the isolation and identification of the *Brucella abortus* were commonly accepted as the gold standard method, it cannot meet the requirements for early diagnostic strategies. Conventional PCR techniques and immunological tests can realize rapid detection of *B. abortus*, but the demands for PCR thermal cyclers and/or specific antibodies hinder their application in basic laboratories. Thus, rapid, sensitive, and specific diagnostic strategies are essential to prevent and control the spread of the bovine brucellosis. In this work, a novel detection method for the rapid identification of *B. abortus*, which uses loop-mediated isothermal amplification (LAMP) combined with a label-based polymer nanoparticles lateral flow immunoassay biosensor (LFIA), was established. One set of specific *B. abortus*-LAMP primers targeting the *BruAb2_0168* gene was designed by the online LAMP primer design tool. The *B. abortus*-LAMP-LFIA assay was optimized and evaluated using various pathogens and whole blood samples. The optimal amplification temperature and time for *B. abortus*-LAMP-LFIA were determined to be 65°C and 50 min, respectively. The *B. abortus*-LAMP-LFIA method limit of detection (LoD) was 100 fg per reaction for pure genomic DNA of *B. abortus*. Meanwhile, the detection specificity was 100%, and there was no cross-reactivity for other *Brucella* members and non-*Brucella* strains. Furthermore, the entire procedure, including the DNA preparation for whole blood samples (30 min), isothermal incubation (50 min), and LFIA detection (2–5 min), can be completed in approximately 85 min. Thus, the *B. abortus*-LAMP-LFIA assay developed was a simple, rapid, sensitive, and reliable detection technique, which can be used as a screening and/or diagnostic tool for *B. abortus* in the field and basic laboratories.

## Introduction

Brucellosis is a worldwide major zoonotic disease caused by members of the genus *Brucella*, with more than half a million new cases reported annually (Hinić et al., 2008; Moeini-Zanjani et al., 2020). It can manifest as undulating fever with arthralgia, sometimes associated with chronic and severe complications (e.g., orchitis, spondylitis, and arthritis) and remains as a common cause of pyrexia of unknown origin (PUO) (Zhong et al., 2013; Daugaliyeva et al., 2018; Patra et al., 2019). Meanwhile, the symptoms of infection in animals include abortion, infertility, and decreased production. *Brucella* spp. are Gram-negative, facultative, and intracellular pathogens with 10 different species-specific host preferences (e.g., *Brucella melitensis*, *Brucella abortus*, *Brucella suis*, *Brucella ovis*, *Brucella canis*, *Brucella neotomae*, etc.) (Zhong et al., 2013; Daugaliyeva et al., 2018). Especially, *Brucella abortus* (*B. abortus*), an important pathogen of the genus *Brucella*, is a major causative agent of abortion and infertility in case of cattle population (namely, bovine brucellosis is an important infectious disease in the cattle population) (Ali et al., 2014; Islam et al., 2019; Khurana et al., 2021). Many countries have adopted different strategies to control this disease from their cattle herd. Thus, the ability to early, rapidly, and specially differentiate *B. abortus* is essential to control the disease.

Currently, isolation and identification of the *Brucella* spp*.* from blood culture are universally regarded as the standard diagnostic methods for laboratory examination (Patra et al., 2019). However, bacteriological isolation and identification methods (e.g., Rose Bengal plate test, serum agglutination test, and phage lysis test) are time-consuming and low sensitivity, and contain a risk of infection for laboratory personnel, so it is difficult to meet the requirements for early strategies (Li et al., 2019a; Khurana et al., 2021). Thus, rapid, safe, and sensitive identification techniques are required for the detection of specific-species of *Brucella* spp. Presently, several rapid detection methods based on immunological tests, including enzyme-linked immunosorbent assay (ELISA) and fluorescence polarization immunoassay (FPIA) have been successfully applied for the serological diagnosis of brucellosis (Xu et al., 2020; Dong et al., 2021). Although the sensitivity of the immunological techniques is between 65 and 95%, the low sensitivity of the acute phase, the low specificity of the antibodies, and the need for expensive reagents are major limitations of the methods (Özdemir et al., 2011; Karthik et al., 2014).

In recent years, various molecular detection techniques for the identification of *Brucella* spp*.* have been developed. Among them, polymerase chain reaction (PCR) and PCR-based assay (i.e., multiplex PCR and real-time PCR) are used as the conventional molecular detection methods in clinical examinations of brucellosis (Hinić et al., 2008; Surucuoglu et al., 2009; Kang et al., 2011). Despite these methods own outstanding analytical capabilities, the shortcomings (special apparatus’ requirements, poor availability, and long detection procedure) restrict their application in the point-of-care and field laboratories (Li et al., 2019a; Yang et al., 2021). To address the shortcomings of PCR-based techniques, loop-mediated isothermal amplification (LAMP), which includes 4 core primers (F3, B3, FIP, and BIP) and/or two loop primers (LF and LB) on target sequences, was developed in 2000 by Notomi et al. (2000). LAMP technique, as a fast, reliable, simple, and sensitive isothermal detection method, has been performed to detect various pathogens (containing bacteria, viruses, fungi, and emerging/re-emerging infectious agents) (Wang et al., 2017; Kashir and Yaqinuddin, 2020; Yang et al., 2021). In previous reports, the LAMP technique has been used to detect *Brucella* ssp*.* in diagnosis assay. Unfortunately, conventional validation methods for LAMP amplicons, including hydroxy naphthol blue (HNB), SYBR Green, and agarose gel electrophoresis, are difficult to accurately distinguish specific amplification from non-specific amplification, which can easily lead to misinterpretation of the results (Li et al., 2019a; Yang et al., 2021).

To overcome these defects, a target-specific, simple, and visual nanoparticle-based lateral flow immunoassay biosensor (LFIA) was successfully designed and applied to verify LAMP products (Li et al., 2019a; Yang et al., 2021). The detection mechanism of LFIA is that LAMP reaction amplicons, which are composed of 6-carboxyfluorescein (6-FAM) and biotin, combined with dye streptavidin-coated polymer nanoparticles (SA-PNPs) to form a complex (6-FAM-biotin-SA-PNPs). Then, after the complex is captured by the rabbit anti-fluorescein antibody (anti-FITC) on the LFIA, the detection for pathogen nucleic acid is realized by the visual test line (Gong et al., 2019; Li et al., 2020). Although LAMP- and MCDA-LFIA (multiple cross displacement amplification) methods targeting *Bscp31*, *mcr-1*, *IS6110*, and *mtp40* gene for detection of *Brucella* spp. (genus level) and/or other target pathogens have been established, these methods cannot accurately detect and/or identify the *Brucella abortus* strains (species level) (Li et al., 2019a; Li et al., 2019b; Gong et al., 2019; Yang et al., 2021). Previously, conventional PCR and LAMP assays targeting *BruAb2_0168* gene were developed and applied to identify the *B. abortus* in accurate diagnosis tests of brucellosis (Hinić et al., 2008; Kang et al., 2015).

In this report, a novel LFIA detector linked to LAMP technique (LAMP-LFIA) was developed and used for the visual, simple, sensitive, and specific identification of *B. abortus* (*B. abortus*-LAMP-LFIA) by highly specific region on the *BruAb2_0168* gene. These improvements overcome the detection complexity of traditional methods and realize the accurate identification of specific-species of *Brucella* spp. (namely *Brucella abortus*). In *B. abortus*-LAMP-LFIA system, a unique-region of *BruAb2_0168* gene was amplified in the reaction mixture, and results were indicated using LFIA. The optimal reaction conditions and feasibility of the *B. abortus*-LAMP-LFIA assay were confirmed by using DNA from pure cultures and whole blood samples.

## Materials and Methods

### Ethical Statement

The study was approved by the Human Ethics Committee of the Guizhou Provincial Center for Disease Control and Prevention and complied with the Declaration of Helsinki. All data/isolates were analyzed anonymously.

### Reagents and Apparatus

Universal DNA isothermal amplification kits and visual malachite green (MG) were provided by Bei-Jing HaiTaiZhengYuan. Co., Ltd. (Beijing, China). Bacterial genomic DNA extraction kits were purchased from Takara Biomedical Technology Co., Ltd. (Beijing, China). Biotin-14-dctp (0.1 mM) was provided by Tian-Jin Huidexin Technology Development Co., Ltd. (Tianjin, China). These materials including backing card, sample pad, conjugate pad, nitrocellulose membrane (NC), and absorbent pad were provided by Jie-Yi Biotechnology. Co., Ltd. (Shanghai, China). Biotinylated bovine serum albumin (biotin-BSA) and anti-FITC were purchased from Abcam. Co., Ltd. (Shanghai, China). Dye (crimson red) streptavidin-coated polymer nanoparticles (SA-PNP) (129 nm, 10 mg ml^−1^, 100 mM borate, pH 8.5 with 0.1% BSA, 0.05% Tween 20, and 10 mM EDTA) were obtained from Bangs Laboratories, INC. (Indiana, United States).

### Preparation of the Nanoparticle-Based Biosensor

**FIGURE 1:**
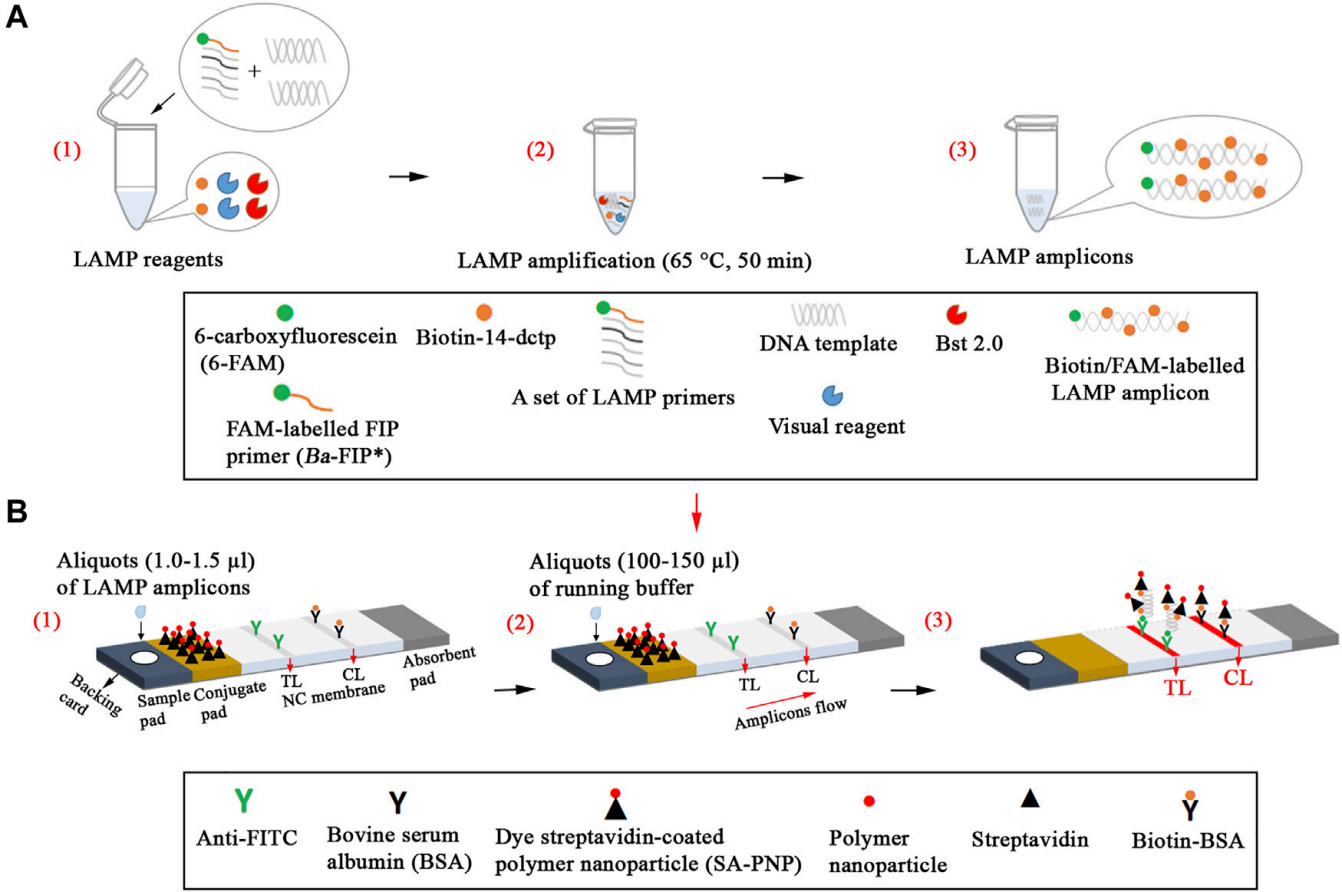
Schematic description of the *B. abortus*-LAMP-LFIA assay. **(A1)**, Preparing the reaction mixtures. **(A2)**, *B. abortus*-LAMP amplification. **(A3)**, The detectable LAMP amplicons after amplification. *B. abortus*-LAMP amplicons were simultaneously labeled with FAM and biotin. **(B1)**, Aliquots (1.0–1.5 µl) of LAMP amplicons was deposited on the sample pad of LFIA. **(B2)**, Aliquots (100–150 µl) of running buffer were also deposited on the sample pad of LFIA. **(** **B3** **)**, The biotin/FAM-labeled amplification products were captured by the anti-fluorescein body (anti-FITC) fixed on the test line (TL) of the biosensor; the surplus SA-PNPs (dye streptavidin-coated polymer nanoparticle, crimson red) were captured by the biotin-BSA (biotinylated bovine serum albumin) fixed on the control line (CL) of the biosensor, which demonstrated the working condition of the biosensor. 6-FAM, 6-carboxyfluorescein; *B. abortus*, *Brucella abortus*; LAMP, loop-mediated isothermal amplification; LFIA, lateral flow immunoassay biosensor.

Nanoparticle-based biosensors (60 × 4 mm) were designed based on a previous publication in our study (Wang et al., 2018). Briefly, the sample pad, conjugate pad, NC membrane, and absorbent pad were attached to a plastic adhesive backing card. Then, the capture reagents, containing anti-FITC (0.15 mg/ml) and biotin-BSA (2.5 mg/ml) in 0.01 M phosphate buffered saline (PBS, PH 7.4), were immobilized on the NC membrane. Thus, there were two bands, including control line (CL) conjugated with biotin-BSA and test line (TL) conjugated with anti-FITC, which were separated by 5 mm. SA-PNPs (129 nm, 10 mg ml^−1^, 100 mM borate, pH 8.5 with 0.1% BSA, 0.05% Tween 20, and 10 mM EDTA) in 0.01M PBS (PH 7.4) were collected in the conjugate pad. The assembled cards were cut into 4-mm wide strips (Deli No. 8012). The assembled biosensors were packaged in a plastic box containing a desiccant gel and stored in a dry and dark place (room temperature). According to our design, the LFIAs were timely manufactured by Tian-Jin HuiDeXin Biotech. Co., Ltd. (Tianjin, China). A schematic description of the *B. abortus*-LAMP-LFIA assay is displayed in Figure 1. An aliquot (1.0–1.5 µl) of LAMP amplicons was deposited on the sample pad of LFIA, and then an aliquot (100–150 µl) of running buffer is also deposited on the sample pad of LFIA in our study.

### Loop-Mediated Isothermal Amplification Primers Design and Screening

**FIGURE 2:**
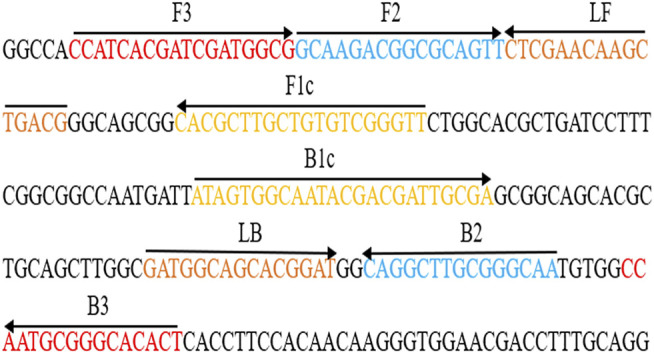
Primers specific to *BruAb2_0168* gene of *B. abortus* used for the *B. abortus*-LAMP-LFIA assay. The sequence and location of the *BruAb2_0168* gene was performed to design *B. abortus*-LAMP primers. Primer *Ba*-FIP contained F1c and F2; primer *Ba*-BIP contained B1c and B2. The direction of arrows indicated the primer from 5′ to 3′. LFIA, lateral flow immunoassay biosensor; LAMP, loop-mediated isothermal amplification.

After sequence alignment and screening using the BLASTn (Basic Local Alignment Search Tool), six primers (*Ba*-FIP, *Ba*-BIP, *Ba*-LF, *Ba*-LB, *Ba*-F3 and *Ba*-B3) for the *BruAb2_0168* gene (GenBank accession no. AE017224.1) was designed via online website (primerexplorer.j*p*/lampv5e/index.html). 6-FAM was labeled on the 5′ end of the *Ba-*FIP primer. The primer information (i.e., sequence, length, and modification) is shown in Figure 2 and Table 1. All primers (HPLC purification grade) used in our study were synthesized and purified by Tianyi-Huiyuan Biotech Co., Ltd. (Beijing, China). More than 4 sets of LAMP primer targeting *BruAb2_0168* gene were designed and synthesized, and they were used to screen the optimal primer set by observing specific-amplification, cross-reactivity, and reaction speed in the current report.

**TABLE 1:** The primers used in the *B. abortus*-LAMP-LFIA assay.

Gene	Primer	Sequence (5′-3′)^a^	Length^b^ (nt)
*BruAb2_0168*	*Ba*-F3	5′- CCA​TCA​CGA​TCG​ATG​GCG-3′	18
	*Ba*-B3	5′-AGT​GTG​CCC​GCA​TTG​G-3′	16
	*Ba*-FIP*	5′-FAM-AACCCGACACAGCAAGCGTGGCAAGACGGCGCAGTT-3′	36
	*Ba*-BIP	5′- ATA​GTG​GCA​ATA​CGA​CGA​TTG​CGA​TTG​CCC​GCA​AGC​CTG -3′	39
	*Ba*-LF	5′-CGT​CAG​CTT​GTT​CGA​G-3′	16
	*Ba*-LB	5′-GAT​GGC​AGC​ACG​GAT-3′	15

a FAM, 6-Carboxyfluorescein.

b nt, nucleotide.

### Bacterial Strains and DNA Extraction

A total number of 16 *Brucella* strains, including *B. abortus* (reference strain 544, isolated strains, vaccine strain A19), *B. melitensis* (reference strain 16M, isolated strains, vaccine strains M5 and M28), *B. suis* (reference strain 1330S, isolated strains, vaccine strain S2), and *B. canis* (isolated strain), and 7 non*-Brucella* isolates were used in this report (Table 2). Then, DNA templates for 23 bacterial strains were prepared using the bacterial genomic DNA extraction kits, and stored at −20°C. The genomic DNA for *B. abortus* 544 was tested at the 260/280 wavelengths using an ultraviolet spectrophotometer, and serial dilutions (1 ng, 100 pg, 10 pg, 1 pg, 100 fg, 10 fg, and 1 fg per microliter) were prepared for primer sets screening, confirmation test, reaction temperature and time optimization, sensitivity, and specificity analysis.

**TABLE 2:** The information of bacterial strains in this study.

Bacteria	Strain no. (source of strains)^a^	No. of strains	LAMP-LFIA result^b^
*Brucella species*			
*B. abortus*	544 (ATCC 23448)	1	P
*B. abortus*	A19 (GZCDC)	1	P
*B. abortus*	2038 (GZCDC)	1	P
*B. abortus*	Isolated strain (GZCDC)	4	P
*B. melitensis*	16M (ATCC 23456)	1	N
*B. melitensis*	M28 (GZCDC)	1	N
*B. melitensis*	M5 (GZCDC)	1	N
*B. melitensis*	Isolated strains (GZCDC)	2	N
*B. suis*	1330S (ATCC 23444)	1	N
*B. suis*	S2 (GZCDC)	1	N
*B. suis*	Isolated strain (GZCDC)	1	N
*B. canis*	Isolated strain (GZCDC)	1	N
*Non-Brucella species*			
*Mycobacterium tuberculosis*	H37Rv (ATCC 27294)	1	N
*Mycobacterium bovis*	ATCC 19210	1	N
*Listeria monocytogenes*	Isolated strain (GZCDC)	1	N
*Salmonella* spp.	Isolated strain (GZCDC)	1	N
*Klebsiella pneumoniae*	Isolated strain (GZCDC)	1	N
*Pseudomonas aeruginosa*	Isolated strain (GZCDC)	1	N
*Streptococcus pneumoniae*	Isolated strain (GZCDC)	1	N
Total		23	—

a ATCC, american type culture collection; GZCDC, guizhou provincial center for disease control and prevention.

b P, positive; N, negative.

### Processing the Whole Blood Samples

A total of 86 whole blood samples, which were suspected from bovine brucellosis, were collected from different regions of Guizhou province, China. All blood samples were divided equally into two parts (Part I and Part II). Whole blood samples (Part I) were cultured using the BACTEC FX system (Becton-Dickinson, Sparks, MD), incubated for 6 weeks and sub-cultured weekly (Sagi et al., 2017). Briefly, the blood samples (approximately 3 ml) collected from cattle that were aseptically inoculated into a two-phase culture flask (BIOVD, Zhengzhou, Henan, China) to cultivate and isolate *Brucella* strains. Post-incubation at the conditions of 37°C with 5% CO_2_ for 3–5 days (or more 3–5 days cultivation for blind passage), the bacteria strain was streaked on blood agar plate and *Brucella* agar plate for pure cultivation. Suspected *Brucella* strains (genus level) were identified based on conventional biochemical tests, Gram staining and serum agglutination tests (Susceptible and Variant, 1990). Subsequent *B. abortus* strains (species level) were further identified using phage lysis tests in *Brucella* isolates according to a previous publication (Susceptible and Variant, 1990). The blood samples (Part II) were subjected to *B. abortus*-PCR and *B. abortus*-LAMP-LFIA assays by using the protocol of QIAamp to directly extract DNA templates from these samples (500 μl) (Li et al., 2019a).

### The Standard Loop-Mediated Isothermal Amplification Reaction

The availability of optimal LAMP primer for *BruAb2_0168* gene was confirmed using the standard LAMP reaction with the follow-up test (Li et al., 2019a). The reaction system (25 μl) of LAMP assay containing the following: 12.5 μl 2 × reaction buffer, 1 μl 2.0 Bst DNA polymerase, 1.6 μM each of FIP* and BIP, 0.8 μM each of LF and LB, 0.4 μM each of F3 and B3, 1 μl of biotin-14-dCTP, 1 μl MG indicator, DNA templates (1 μl of pure culture and 4 μl of samples), and double distilled water (ddH_2_O) were added to 25 μl. The reaction tubes were incubated at 63°C for 60 min and then it was terminated at 85°C for 5 min. Finally, the LAMP amplicons were verified using nanoparticle-based biosensor, MG reagents, and real-time turbidimeter *LA-*500 (Eiken Chemical Co., Ltd. Japan).

### Optimization of Reaction Temperature and Time

Then, the optimal temperature of the *B. abortus*-LAMP-LFIA assay was confirmed by setting the different reaction temperature (63–70°C, intervals 1°C), and the template DNA (10 pg/μl) of *B. abortus* 544 was employed in this study. The assay was performed according to the standard LAMP assay and monitored using the turbidimeter. In addition, the threshold value (turbidity) was 0.1, and a turbidity of >0.1 was considered as positive amplification (Li et al., 2019a).

Moreover, the effect of different times (10–60 min, with 10 min intervals) on *B. abortus*-LAMP-LFIA assay was evaluated, and the amplicons were detected with biosensors. When MG reagent was added to the reaction mixtures, the color of reaction tubes for LAMP positive amplification changed from dark blue to blue, while the negative control and blank control were light blue or colorless. In the experiment, 1 μl genomic DNA of *Listeria monocytogenes* and/or *Mycobacterium tuberculosis* was used as negative control (NC) and 1 μl of ddH_2_O was used as blank control (BC).

### Sensitivity and Specificity of the *B. abortus*-LAMP-LFIA Assay

The sensitivity of the *B. abortus*-LAMP-LFIA assay was confirmed using serial dilutions (1 ng/μl -1 fg/μl) of the template DNA of *B. abortus* 544, and the test was performed using a defined number of replicates (usually 20 per dilution). The limit of detection (LoD) of the LAMP-LFIA was defined as the lowest concentration of genomic DNA that, when detected by serial dilutions, resulted in the detection of *B. abortus* in ≥95% of the assays conducted in the current research (Chakravorty et al., 2017). The results of sensitivity assays were reported by LFIAs, MG indicator, and turbidimeter, and all tests were independently implemented in multiple replicates.

In order to evaluate the specificity of *B. abortus*-LAMP-LFIA assay, genomic DNA of 23 bacterial strains was detected according to the optimal amplification temperature and time (Table 2). The assay results were verified using LFIAs.

### Applicability of the *B. abortus*-LAMP-LFIA Assay to Whole Blood Samples

In order to evaluate the practicability of *B. abortus*-LAMP-LFIA assay for *B. abortus* detection, 86 samples were tested by culture-biotechnical methods (i.e., Gram staining, biochemical tests, serum agglutination tests, and phage lysis tests), *B. abortus*-PCR, and *B. abortus*-LAMP-LFIA assay. According to the abovementioned extraction steps of genomic DNA from whole blood samples, 4 μl of template DNA was used for *B. abortus*-PCR and *B. abortus*-LAMP-LFIA tests. *B. abortus*-PCR tests were performed with reference to previous publications (Hinić et al., 2008), and the reaction mixtures (25 μl) contained the following: 12.5 μl 2 × Taq Master Mix (CoWin Biosciences Co., Ltd. Beijing, China), 0.2 μM *Ba*-F (5′-TCG-CAT-CGG-CAG-TTT-CAA-3′), 0.2 μM *Ba*-R (5′-CCA-GCT-TTT-GGC-CTT-TTC-C-3′), 4 μl of samples DNA, and ddH_2_O was added to 25 μl. The reactions were carried out using an automated thermal cycler (Thermo Fisher Scientific Co., Ltd. Beijing, China). The reaction mixtures were denatured at 94°C for 2 min, and 35 reaction cycles were conducted. The cycles consisted of denaturation at 94°C (30 s), annealing at 59°C (30 s), and primer extension at 72°C (30 s). The final extension time was set for 2 min. The PCR products were visualized in a 2.0% agarose gel with GelRed staining under UV light (BioRad, United States). The *B. abortus*-LAMP-LFIA assay was applied according to optimal reaction conditions.

### Statistical Analysis

In this report, the results of 86 whole blood samples tested by culture-biotechnical methods (as the standard method) were used as the standard, and they were further analyzed and calculated for sensitivity (%), specificity (%), positive predictive value (PPV%), and negative predictive value (NPV%) of *B. abortus*-PCR and *B. abortus*-LAMP-LFIA methods using SPSS software (ver. 26.0; IBM, United States).

## Results

### Confirmation and Validation of *B. abortus*-LAMP Amplicons

**FIGURE 3:**
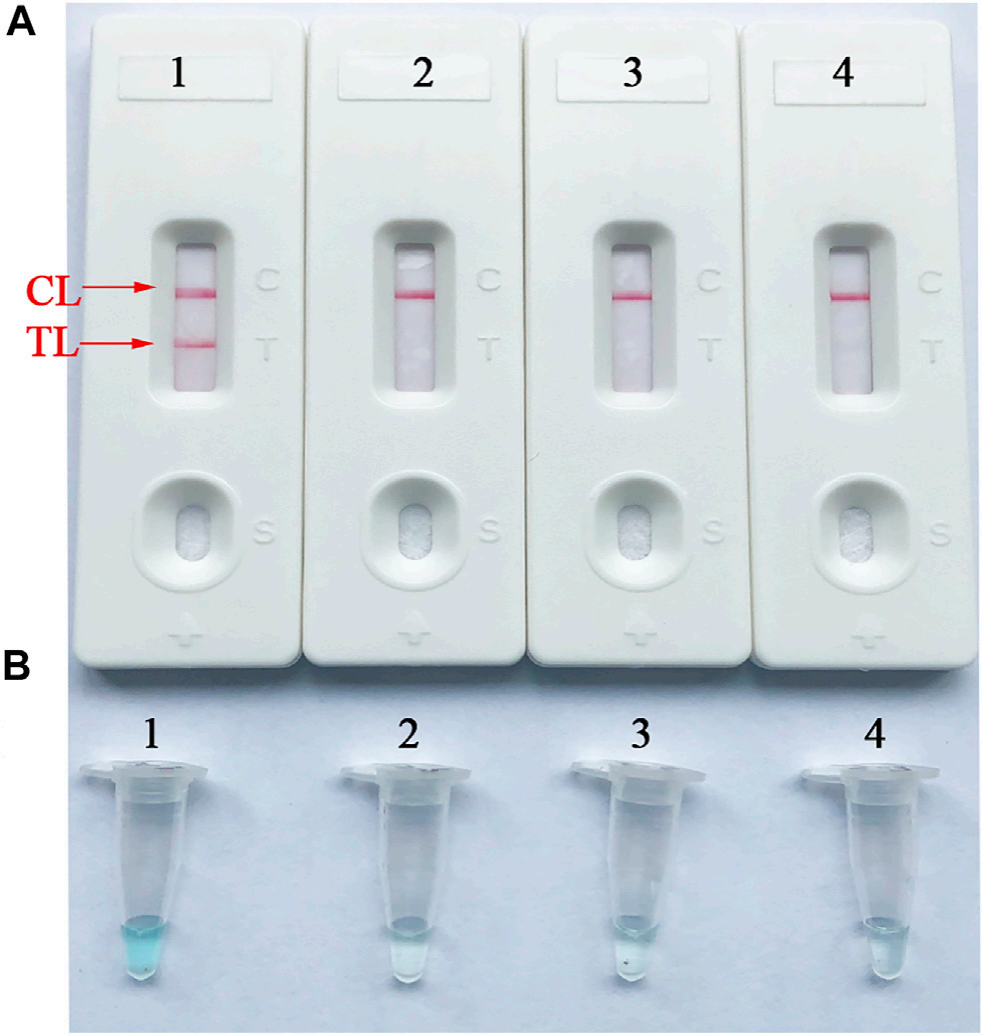
Verification and analysis of *B. abortus*-LAMP-LFIA amplicons. **(A)** The LFIA strips used for rapid detection of *B. abortus*-LAMP-LFIA products. **(B)** Color change for *B. abortus*-LAMP-LFIA tubes. Strip/tube 1: positive reaction of *B. abortus-*LAMP products for the *B. abortus* 544; strip/tube 2: negative control (*Listeria monocytogenes*); strip/tube 3: negative control (*Mycobacterium tuberculosis*); strip/tube 4: blank control (ddH_2_O). LAMP, loop-mediated isothermal amplification; LFIA, lateral flow immunoassay biosensor; TL, test line; CL, control line; ddH_2_O, double distilled water.

To confirm the availability of an optimal primer set screened from more than 4 primer sets, *B. abortus*-LAMP tests were performed using the genomic DNA (10 pg/μl) of *B. abortus* 544. The CL (control line) and TL (test line) were red for positive amplification, while only the CL line was red for negative and blank control (Figure 3A). Meanwhile, the positive amplification tubes changed from dark blue to blue, while the negative tubes were colorless (Figure 3B).

### Optimal Reaction Temperature and Time for *B. abortus*-LAMP-LFIA Assay

**FIGURE 4:**
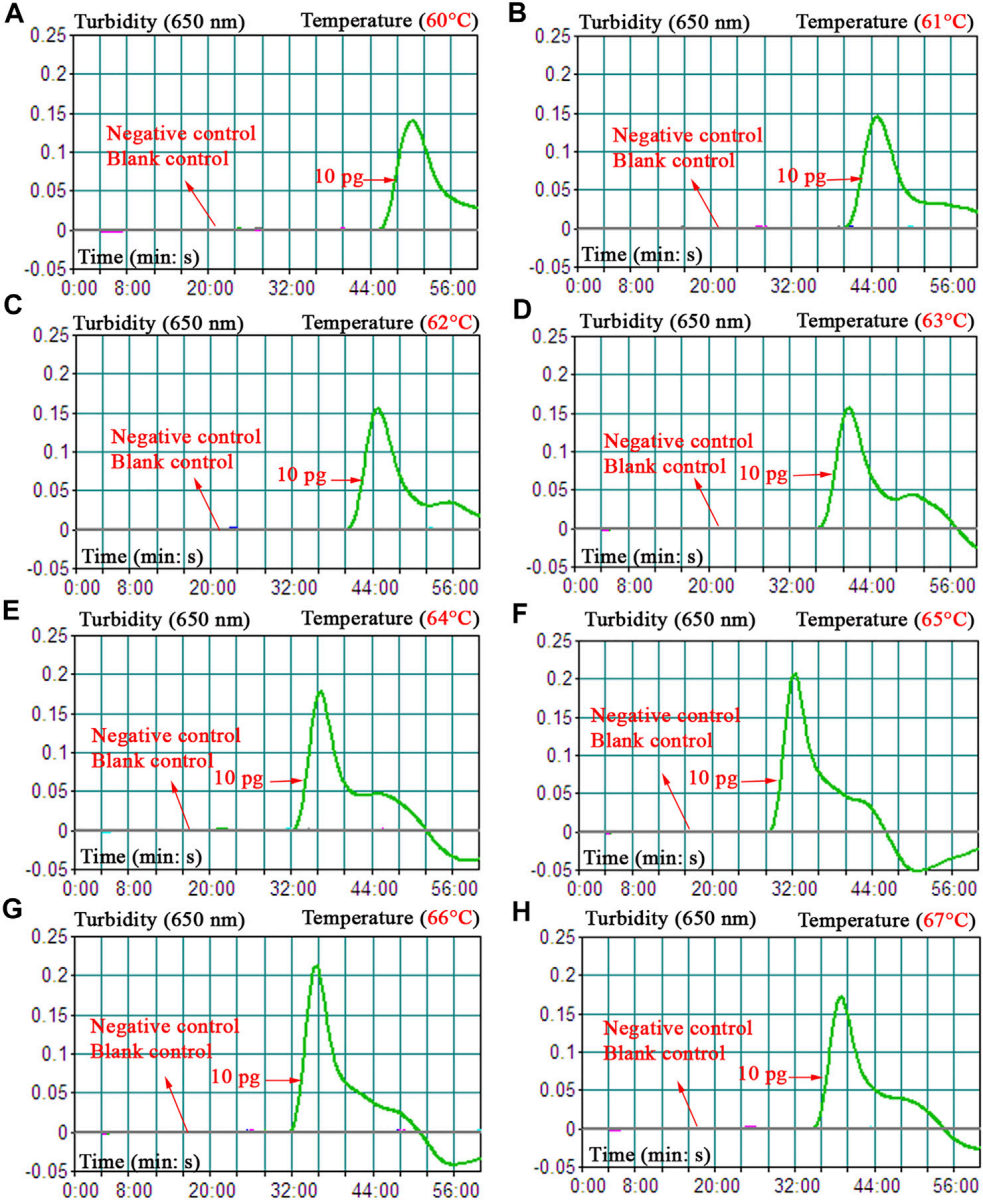
Optimization of amplification temperature for the *B. abortus*-LAMP-LFIA assay. The standard *B. abortus*-LAMP assays were monitored by real-time turbidimetry, and the corresponding information was marked in the drawings. This threshold value was 0.1, and a turbidity >0.1 was judged as a positive reaction. A total of eight kinetic graphs **(A–H)** were generated at various temperatures (60–67°C, 1°C intervals) with template DNA at the level of 10 pg/μl. The drawings from **(E–G)** showed higher amplification efficiency. LAMP, loop-mediated isothermal amplification; LFIA, lateral flow immunoassay biosensor; TL, test line; CL, control line; ddH_2_O, double distilled water; BC, blank control.

To obtain the optimal reaction temperature for the *B. abortus*-LAMP-LFIA experiment, the optimization test was performed by setting a series of amplification temperatures (60–67°C, with 1°C interval). Among the eight kinetics graphs generated, the *B. abortus*-LAMP-LFIA assay exhibited higher amplification efficiency when the test temperature ranged from 64 to 66°C (Figure 4).

Different incubation times (ranging from 10 to 60 min, with 10-min intervals) were applied to verify the optimal reaction time for the *B. abortus*-LAMP-LFIA assay. Time optimization tests demonstrated that the biosensors could detect the genomic DNA of *B. abortus* 544 at a minimum concentration of 100 fg/μl when the reaction time was 50–60 min (Table 3). Thus, a reaction temperature of 65°C and an amplification time of 50 min were used as the optimal conditions for the rest of the *B. abortus*-LAMP-LFIA assays in the current study.

**TABLE 3:** The optimization of amplification time for the *B. abortus*-LAMP-LFIA assay.

Time/min	Serial dilutions of genomic DNA (*B. abortus* 544)							
	1 ng	100 pg	10 pg	1 pg	100 fg^a^	10 fg	1 fg	BC^b^
10	−	−	−	−	−	−	−	−
20	+	+	−	−	−	−	−	−
30	+	+	+	+	−	−	−	−
40	+	+	+	+	+/−	−	−	−
50	+	+	+	+	+	−	−	−
60	+	+	+	+	+	−	−	−

+ positive amplification; -, negative amplification.

a +/-, weak positive amplification.

b BC, blank control.

### Detection Sensitivity of *B. abortus*-LAMP-LFIA Assay

**FIGURE 5:**
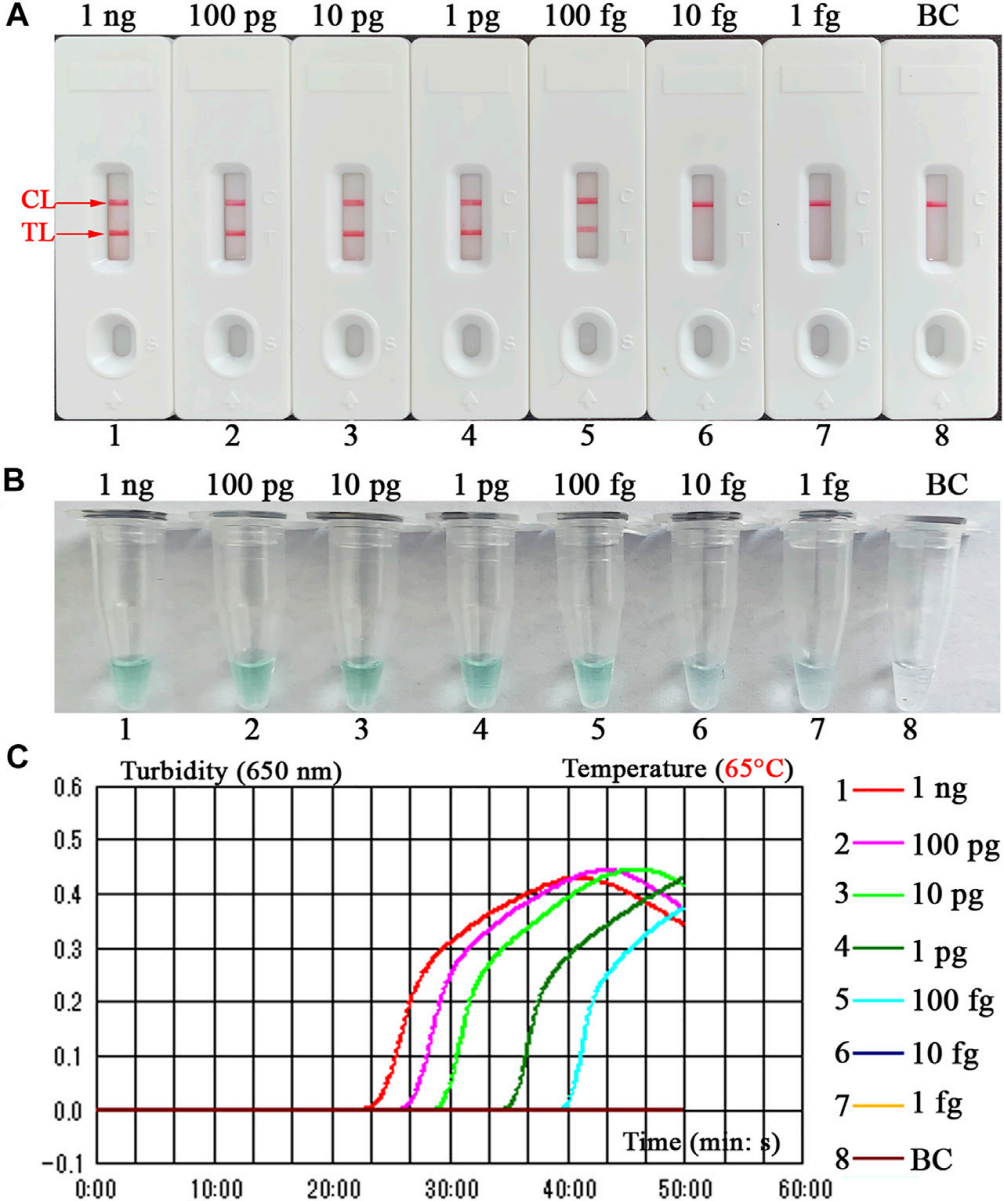
Detection sensitivity for the *B. abortus*-LAMP-LFIA assay. The experiments were performed according to the optimal reaction condition, and serial dilutions (1 ng, 100 pg, 10 pg, 1 pg, 100 fg, 10 fg, and 1 fg per microliter) of target DNA template were tested. Three validation tools, containing LFIA strips **(A)**, MG reagents **(B)**, and real-time turbidity **(C)**, were used to verify the *B. abortus-*LAMP amplification products. Strips **(A)**/tubes **(B)**/curves **(C)** 1-7 correspond to DNA template of *B. abortus* 544 from 1 ng/μl to 1 fg/μl, strip/tube/curve 8: blank control (ddH_2_O). MG, malachite green; LAMP, loop-mediated isothermal amplification; LFIA, lateral flow immunoassay biosensor; ddH_2_O, double distilled water; TL, test line; CL, control line. All tests were independently implemented in multiple replicates.

The sensitivity of *B. abortus*-LAMP-LFIA method was confirmed by repeated detection for serial dilutions of genomic DNA of *B. abortus* 544 in our study. The *B. abortus*-LAMP-LFIA assay LoDs were 100 fg of template DNA per microliter. TL and CL lines (red) could be observed on LFIA, indicating positive amplification for *BruAb2_0168* gene (Figure 5A). In particular, LFIA verification results for LAMP amplicons were consistent with MG visual indicators (Figure 5B) and real-time turbidity (Figure 5C).

### Detection Specificity of *B. abortus*-LAMP-LFIA Assay

**FIGURE 6:**
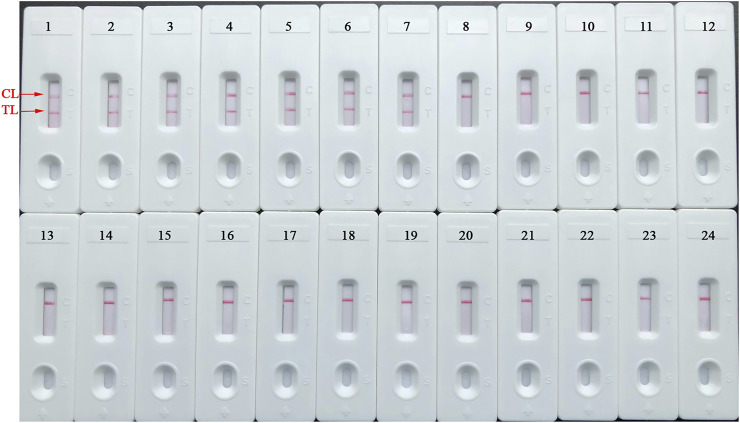
Detection specificity of *B. abortus*-LAMP-LFIA assay for various pathogens. The *B. abortus*-LAMP tests were performed using template DNA of different pathogens, and the amplicons were verified by LFIA strips. Strips 1–3, *B. abortus* 544 (ATCC 23448), *B. abortus* A19, and *B. abortus* 2038; strips 4–7, *B. abortus* (isolates); strips 8–10, *B. melitensis* 16M (ATCC 23456), *B. melitensis* M28, and *B. melitensis* M5; strips 11–12, *B. melitensis* (isolates); strips 13–16, *B. suis* 1330S (ATCC 23444), *B. suis* S2, *B. suis* (isolate), and *B. canis* (isolate); strips 17–23, *Mycobacterium tuberculosis*, *Mycobacterium bovis*, *Listeria monocytogenes*, *Salmonella* spp., *Klebsiella pneumoniae*, *Pseudomonas aeruginosa,* and *Streptococcus pneumoniae*; strip 24, blank control (ddH_2_O). LAMP, loop-mediated isothermal amplification; LFIA, lateral flow immunoassay biosensor; TL, test line; CL, control line; ddH_2_O, double distilled water; BC, blank control; ATCC, American Type Culture Collection.

The detection specificity of LAMP-LFIA was evaluated using genomic DNA extracted from 23 bacterial strains (i.e., *Brucella* species/strains and non*-Brucella* strains) (Table 2). The analysis specificity of *B. abortus*-LAMP-LFIA was 100% in our study. The *B. abortus*-LAMP-LFIA method can specifically detect representative strains of *B. abortus* (isolates, reference, and vaccine strains), but other bacterial pathogens (other *Brucella* members and non*-Brucella* strains) cannot be detected (Figure 6).

### Practical Evaluation of the *B. abortus*-LAMP-LFIA Assay for Whole Blood Samples

A total of 86 whole blood samples were tested by conventional culture-biotechnical methods, *B. abortus*-PCR, and *B. abortus*-LAMP-LFIA assay as described above. Then, the practicability of *B. abortus*-LAMP-LFIA assay was further evaluated by comparing with the results of culture-biotechnical methods and *B. abortus*-PCR assay. In the whole blood sample detection results, 9 samples were tested as positive, and 77 samples were negative by the culture-biotechnical methods. Nine were detected as positive and 77 were negative by *B. abortus*-LAMP-LFIA assay. However, only 7 samples were examined as positive, and 79 samples were negative by *B. abortus*-PCR method. Both the sensitivity and specificity of *B. abortus*-LAMP-LFIA was 100% (based on the standard results of culture-biotechnical methods) (Table 4). These results show that the *B. abortus*-LAMP-LFIA assay developed in the current study was a valuable diagnostic tool to detect whole blood samples.

**TABLE 4:** Comparison of culture-biotechnical, *B. abortus*-PCR and *B. abortus*-LAMP-LFIA assay for the detection of *B. abortus* in whole blood samples.

Detection methods^a^	Culture-biotechnical		Sensitivity (%)	Specificity (%)	PPV^b^ (%)	NPV^c^ (%)
	Positive (N = 9)	Negative (N = 77)				
*B. abortus*-PCR						
Positive	7	0	77.78	100	100	97.47
Negative	2	77				
*B. abortus-*LAMP-LFIA						
Positive	9	0	100	100	100	100
Negative	0	77				

a *B. abortus*, *Brucella abortus*; PCR, polymerase chain reaction; LAMP, loop-mediated isothermal amplification; LFIA, lateral flow immunoassay biosensor.

b PPV, positive predictive value; PPV = (true positive/true positive + false positive) * 100.

c NPV, negative predictive value; NPV = (true negative/true negative + false negative) * 100.

## Discussion

Currently, although the isolation and identification (namely culture-biotechnical methods) of the *Brucella* spp. were commonly accepted as the gold standard method, these deficiencies (e.g., time-consuming, complicated operation steps and the risk of infection to laboratory personnel) cannot meet the requirements for early strategies (Schwarz et al., 2017; Li et al., 2019a). However, conventional PCR techniques (single PCR, multiplex PCR, and real-time PCR) and immunological tests (ELISA and FPIA) can realize more rapid detection of *Brucella* spp. than the culture-biotechnical methods, but the demands for PCR thermal cyclers and/or specific antibodies hinder their development in basic laboratories (Surucuoglu et al., 2009; Kang et al., 2011; Xu et al., 2020; Dong et al., 2021). Thus, it is extremely necessary to develop newly diagnostic methods that can meet the above-mentioned requirements.

LAMP, as a low-cost, fast, simple, and efficient nucleic acid amplification technique, seems to be more satisfactory. Presently, LAMP- and LAMP-based assay have been applied in the detection of various pathogens (containing viruses, bacteria, and fungi) (Sharma et al., 2016; Gomes et al., 2020). However, conventional validation tools for LAMP amplicons, including visual indicators, agarose gel electrophoresis, and real-time turbidimeter, are difficult to achieve simple, rapid, and accurate detection of target pathogens (Li et al., 2019a; Yang et al., 2021). A label-based polymer nanoparticle biosensor was developed and applied to specifically verify LAMP amplification products by labeling special primer (*Ba*-FIP primer was labeled 6-FAM) (Li et al., 2019b; Yang et al., 2021). The test results can be verified visually (approximately 2 min) by observing the color of the TL line (red and/or colorless) on the LFIA strips. Although LAMP- and MCDA-LFIA methods targeting *Bscp31* gene for detection of *Brucella* spp. (genus level) have been established in previous publications, these two assays cannot accurately identify the *B. abortus* strains (species level) (Li et al., 2019b; Li et al., 2019a). Meanwhile, conventional LAMP techniques targeting the *BruAb2_0168* gene have achieved rapid detection of *B. abortus* and demonstrated reliable specificity and sensitivity, the shortcomings of frequently used confirmation methods for LAMP amplicons are still unavoidable in a previous work (Karthik et al., 2014). Thus, the nanoparticle biosensor combined with the LAMP technique targeting *BruAb2_0168* gene (*B. abortus*-LAMP-LFIA) has been successfully established and performed to achieve rapid and accurate detection of *B. abortus* in the current report.

A set of unique LAMP primers specifically recognized 8 regions on the *BruAb2_0168* sequence, thus showing high selectivity for the diagnosis of *B. abortus* strains (Figure 2). In order to confirm the optimal reaction conditions of LAMP-LFIA to obtain more efficient amplification, different reaction temperatures (60–67°C, 1°C interval) and times (10–60 min, 10-min intervals) were carried out in our study. When the reaction condition was at 65°C for 50 min, *B. abortus*-LAMP-LFIA assay showed relatively stable amplification (Figure 4 and Table 3). Meanwhile, the specificity of *B. abortus*-LAMP-LFIA assay was 100%, which can not only detect all representative *B. abortus* strains (i.e., reference strains, vaccine strain, and isolates), but also exclude other *Brucella* species (including *B. melitensis*, *B. suis,* and *B. canis*) and non-*Brucella* strains (Figure 6).

In addition, *B. abortus*-LAMP-LFIA showed excellent detection sensitivity and could be detected when the genomic DNA concentration of *B. abortus* 544 was 100 fg per microliter (Figure 5). The sensitivity of the novel nanoparticle-based biosensors combined with LAMP assay was tenfold higher than that of PCR assay (Alamian et al., 2019; Moeini-Zanjani et al., 2020). In follow-up studies, after appropriately extending the amplification time (70–80 min), we found that *B. abortus*-LAMP-LFIA assay can detect the genomic DNA concentration of *B. abortus* 544 at about 80 fg per reaction (data not shown). In addition to using LFIAs to verify LAMP products, we also added MG amplification indicator to the reaction mixture in the experiment, and the results of amplification can be preliminarily judged with the naked eye. As expected, compared with the verification results of LFIAs, positive and negative amplifications do not seem to be clearly distinguished by visual indicators (e.g., the color change between 100 fg and 10 fg reaction tubes). However, the LAMP reaction is prone to aerosol contamination, so the whole experiment was performed in different laboratories (e.g., sample preparation, premixed reaction mixture, LAMP amplification, and LAMP amplicons verification).

In this study, in order to evaluate the practicality of *B. abortus*-LAMP-LFIA in practical examination, a total of 86 whole blood samples collected from suspected bovine brucellosis were tested using culture-biotechnical, conventional *B. abortus*-PCR, and *B. abortus*-LAMP-LFIA methods. The accurate diagnosis efficiency of *B. abortus*-LAMP-LFIA was consistent with the culture-biotechnical methods (Table 4). In addition, ROC analysis (AUC 1.000, the data not shown) based on the sensitivity and specificity of *B. abortus*-LAMP-LFIA was further carried out in our study using the R package (ver. 4. 0. 5). Interestingly, we found that two samples, which were tested as positive by *B. abortus*-LAMP-LFIA and culture-biotechnical, were detected as negative by *B. abortus*-PCR assay. The reasons for the different results are as follows: (i) the concentration of template DNA in the whole blood sample is low and cannot reach the minimum detection limit of *B. abortus*-PCR methods (PCR assay LoDs are usually 1–100 pg per microliter for genomic DNA) (Moeini-Zanjani et al., 2020). (ii) This phenomenon may be caused by false-positive amplification of *B. abortus*-LAMP-LFIA, and the non-specific amplification of conventional LAMP assay was occasionally reported in a previous publication; however, the possibility of false-positives for *B. abortus*-LAMP-LFIA is extremely low, due to these novel strategies were performed (including the specific labeling of primers, the design and application of LFIA biosensors, and the confirmation of conventional culture-biotechnical methods) (Li et al., 2019a; Yang et al., 2021). Meanwhile, 86 blood samples were also examined by the *Brucella* spp.-LAMP-LFIA assay established in our previous experiments (Li et al., 2019a), and the positive results were consistent with *B. abortus*-LAMP-LFIA (data not shown). These data indicate that although the *B. abortus*-LAMP-LFIA method is as sensitive as the culture-biotechnical methods for the detection of practical whole blood samples in the current experiment, *B. abortus*-LAMP-LFIA is simpler, faster, safer, and more applicable than the culture-biotechnical methods.

Moreover, compared to other verification tools for LAMP amplicons (including color indicator, real-time turbidity, and agarose gel electrophoresis), nanoparticle-based LFIA is more convenient (amplification products and running buffer are added to the sample pad), visual (the results are determined by directly observing the color changes of CL and TL line), and highly specific (the *Ba*-FIP primer is specifically labeled). Meanwhile, the total cost of a single *B. abortus*-LAMP-LFIA reaction is about 5 USD, including LAMP isothermal reagents (approximately 1.5 USD), LFIA biosensor (approximately 2.5 USD), DNA extraction reagents (approximately 0.2 USD), MG indicator reagents (approximately 0.3 USD), and other reagents and/or materials (approximately 0.5 USD).

## Conclusion

In this report, a simple, visual, and reliable nanoparticle-based LFIA, which can eliminate the use of special equipment and simplify the detection procedure, was newly designed and applied in the current report. The *B. abortus*-LAMP-LFIA assay (*B. abortus-*LAMP combined with a nanoparticle-based LFIA) targeting the *BruAb2_0168* gene was successfully established and performed, and the technique showed excellent sensitivity and specificity for the detection of bacterial strains (including reference strains, vaccine strains, and isolates) and whole blood samples. Hence, the *B. abortus*-LAMP-LFIA test developed was a simple, rapid, sensitive, and reliable detection technique, which can be used as a screening and/or diagnostic tool for *B. abortus* in the field and basic laboratories.

## Data Availability

The raw data supporting the conclusion of this article will be made available by the authors, without undue reservation.

## Abbreviations

ATCC, American type culture collection; *B. abortus, Brucella abortus*; CL, control line; ddH2O, double distilled water; FAM, carboxy fluorescein; GZCDC, Guizhou Provincial Center for Disease Control and Prevention; LAMP, loop-mediated isothermal amplification; LFIA, lateral flow immunoassay biosensor; LAMP-LFIA, loop-mediated isothermal amplification linked to a nanoparticle-based lateral flow immunoassay biosensor; LoD, limit of detection; MG, malachite green; nt, nucleotide; PCR, polymerase chain reaction; TL, test line.
